# Brief Alcohol Interventions With Older Adults: Results of a Systematic Review of Literature

**DOI:** 10.1093/geroni/igab046.870

**Published:** 2021-12-17

**Authors:** Catherine Lemieux, Gregory Purser

**Affiliations:** Louisiana State University, Baton Rouge, Louisiana, United States

## Abstract

Older persons are especially vulnerable to the negative effects of alcohol misuse. National reports show that the older-adult population is the least likely group to perceive a need for treatment and be screened for alcohol-related problems. Little research has examined the impact of brief interventions on different drinking outcomes in at-risk older adults. To address this gap, the current study sought to systematically review empirical literature examining the effectiveness of brief alcohol interventions (BAI) implemented with adults (≥50) engaged in at-risk drinking. The authors developed specific a priori inclusion criteria (e.g., alcohol-related outcome measures, randomized controlled trials, RCT) before beginning the search process. Key terms were entered into 9 databases to yield an initial pool of 5,909 articles, from which 5,572 were excluded. A total of 337 articles remained, from which an additional 89 were excluded. Next, the authors independently reviewed 248 full-text, empirical articles and subsequently excluded 237 that did not satisfy inclusion criteria. Thus, the current systematic review yielded 11 studies representing RCT or experimental designs that employed random assignment. Findings of the review indicated that 7 (63.6%) studies showed a positive effect, with only 1 showing no positive effect of the intervention. For the remaining 3 (27.2%), the positive effect of the intervention was not conclusively determined due to study design issues. Overall findings suggest that BAI are effective in reducing alcohol consumption in the older-adult population. Additional evidence is needed to further knowledge consistent with recent initiatives (e.g., Age-Friendly Health Systems, 4Ms) that promote healthy aging.

